# Ethical issues and the role of the ethics committees during COVID-19 research in pandemic era: a focus on an Italian ethics committee

**DOI:** 10.3389/fpubh.2025.1537863

**Published:** 2025-05-26

**Authors:** Lucia Tattoli, Barbara Abenante, Paolo Cavallo Perin, Marcello Maddalena, Francesco Lupariello

**Affiliations:** ^1^Section of Legal Medicine, University of Turin, Turin, Italy; ^2^Legal Medicine Unit of the Azienda Sanitaria Locale Torino 3 (ASLTO3), Rivoli, Italy; ^3^Department of Medical Sciences, University of Turin, Turin, Italy; ^4^Court of Turin, Turin, Italy

**Keywords:** COVID-19, ethics committees, research, SARS-CoV2 pandemic, ethics

## Abstract

Independent Research Ethics Committees (RECs) are responsible for protecting the rights and safety of participants involved in research studies. They also promote the values of research ethics and ensure the quality of clinical studies. In emergencies, we expect a significant increase in research activities but it is crucial to maintain both the quality of studies and respect for participants’ rights. At the onset of the SARS-CoV-2 pandemic, the World Health Organization recommended an “expedited” approach to REC approvals. This was intended to streamline and optimize review procedures to prevent delays in research that is critical for responding to the global emergency. The authors evaluated the activity of the Inter-company Ethics Committee (IEC) of Turin, Italy, from January 2020 to December 2022 comparing COVID-19-related protocols with those that were not related to COVID-19. Statistical analyses were applied to find if there were statistically significant differences in variables’ distributions between the two groups of studies. The characteristics of the protocols (total 1,667), including study design, funding, and enrollment of minors, were analyzed. Statistical differences were found for three variables: study type, financial support, and distribution of revised protocols by applicants’ medical specialties. The findings underscore the need for careful attention to ethical principles during emergencies, especially given the large number of projects reviewed by the EC. Various challenges were faced, including the demand for expedited approval of proposed studies, the necessity of recognizing the social value of COVID-19 studies while ensuring proper planning and scientific validity, the need to review studies unrelated to COVID-19, and the obligation to protect the dignity and rights of research participants. It is essential to ensure that the standards for ethical review remain uncompromised.

## 1 Introduction

Directive 2001/20/EC of the European Parliament and of the Council, dated 4 April 2001, addresses the approximation of laws, regulations, and administrative provisions among Member States related to the implementation of good clinical practice in clinical studies for medicinal products intended for human use. This directive defines the Ethics Committee (EC) as an independent body composed of health and non-medical professionals. The EC is responsible for ensuring the protection of the rights, safety, and well-being of participants involved in clinical studies (1).

The EC provides a public guarantee of this protection by issuing opinions on various aspects, such as the trial protocol, the suitability of investigators, the facilities used, and the methods and documents that inform participants prior to obtaining their consent (2–4). Additionally, the EC expresses opinions on diagnostic and therapeutic protocols, including experimental ones, makes recommendations, and is dedicated to training health professionals on bioethical subjects (1, 3).

The Declaration of Helsinki, developed by the World Medical Association, outlines the ethical principles for medical research involving human subjects. It starts with the fundamental principle of “primum non nocere,” meaning “first, do no harm.” According to this declaration, physicians must prioritize the best interests of their patients. This approach is essential in medical research involving humans, as the potential benefits must outweigh the risks, and ensuring patients’ health is the top priority. The Declaration marked a significant step in the development of what are now known as Research Ethics Committees (RECs) (3, 5, 6).

The scientific protocols must be submitted to the REC for approval, accompanied by a comprehensive evaluation that is qualified and independent of any influence, while considering the laws and ethical standards regarding participant enrollment (6, 7).

The two additional international milestones are the Oviedo Convention on Human Rights and Biomedicine in 1997, specifically Protocol III, Chapter III, Articles 9 to 12, and the Universal Declaration on Bioethics and Human Rights (UDBHR), Article 19, in 2005. Both documents emphasize the protection of human dignity and identity, as well as the importance of respecting human life within the biomedical field (2, 5, 8).

The principles that define the scientific validity of biomedical research are fundamentally centered on two key aspects: (1) the respect for the rights, safety, and dignity of human subjects, and (2) the reliability and strength of the data derived from the study (3, 9). These principles must always be upheld, as scientific research is a crucial element for the development and progress of every country.

In recent years, ethical considerations have become increasingly significant due to heightened attention to bioethical issues in healthcare, greater awareness of patient autonomy, and the emergence of technological advancements that foster new hopes while also raising new questions (10).

In recent decades, RECs have undergone significant changes due to the full implementation of the European Union Clinical Trials Regulation 536/2014 (EU CTR) on January 31, 2022. This regulation introduced new requirements for submitting and approving interventional clinical trials in the European Union. The implementation of this regulation emphasized the need for uniform procedures for the submission, assessment, and monitoring of protocols across the EU (9, 11–14).

The importance of adhering to ethical rules became even more critical during the COVID-19 pandemic, which initially emerged in Wuhan in January 2020 and subsequently spread across the globe (15).

In the aim to underline the challenges faced in the pandemic era, the authors evaluated the activity of the Inter-company Ethics Committee (IEC) of Turin, Italy, from January 2020 to December 2022 comparing COVID-19-related protocols with those that were not related to COVID-19.

## 2 The challenges of research ethics committees during COVID-19 pandemic

In Italy, as in many other countries, the pandemic prompted the introduction of new decrees aligned with Regulation 536/2014 (EU CTR) (5).

In March 2020, the Italian Medicines Agency (AIFA) issued guidelines regarding the management of clinical trials in Italy during the COVID-19 emergency. These guidelines were in line with provisions set forth by the Decrees of the Italian President of the Council of Ministers aimed at urgent measures for containing and managing the epidemiological emergency. Smart working practices were implemented to continue clinical trial activities, and the submission procedures were simplified. ECs were permitted to evaluate clinical trials remotely, as well as manage clinical trial activities outside investigational sites, including the management of investigational medicinal products, clinical examinations, and monitoring (16).

From March 2020 onward, a series of law decrees (17, 18) delegated the evaluation of all clinical trials involving medicinal products related to COVID-19 to a preliminary assessment by the AIFA Technical Scientific Committee (CTS), followed by authorization from the AIFA Clinical Trial Office. The EC of the National Institute for Infectious Diseases Lazzaro Spallanzani (INMI Spallanzani) in Rome, acting as a national single EC, evaluated the clinical trials and provided a national opinion based on the AIFA CTS’s evaluation on COVID-19-related research. AIFA established a “fast track” process for the online submission of research documentation and requests for clinical trial authorization related to COVID-19 treatment. Applicants were allowed to postpone the submission of paper documents, which still needed to be sent to the CTS as soon as possible.

During the COVID-19 public health emergency, applications for trials in oncology, transplants, and urgent clinical conditions requiring immediate interventions were accepted, in addition to trials addressing the COVID-19 emergency.

The challenge for REC during this exceptional era is to maintain high ethical standards in research while balancing the urgent need to quickly gather evidence for public health interventions with the protection of individuals. It is important to uphold the ethical principles that govern biomedical research (19, 20).

In Italy, the National Institute of Health (ISS), which serves as the technical-scientific body of the Italian National Health Service, emphasized that researchers and ethics committees involved in observational and epidemiological research must consider ethical values and principles during the COVID-19 emergency (8). In October 2020, the Italian Committee for Bioethics published a report addressing the most significant ethical issues related to the experimentation of new treatments, particularly during a time of the pandemic when a vaccine was still being developed (21).

## 3 The experience of the inter-company ethics committee (IEC) of Turin, Italy

The Inter-company Ethics Committee (IEC), instituted in 2019, includes three Hospitals in Turin: the University Hospital “Città della Salute e della Scienza,” Hospital “Ordine Mauriziano” and “ASL Città di Torino.” The IEC is composed of 24 members: 4 clinicians experts on internal and specialistic medicine (1 nephrologist, 1 infectious disease specialist, 1 endocrinologist, 1 expert in rare diseases), 1 surgeon, 1 general practitioner, 1 pediatrician, 1 biostatistics, 2 clinical pharmacologists; 1 legal expert (Chair), 1 forensic pathologist, 1 bioethicist, 1 lay member (representative of patients), 1 representative of the healthcare professions, 1 expert in medical devices, 1 clinical engineer, 1 clinician expert on nutrition (vice-Chair), 1 clinician expert on new techniques, 1 geneticist, 1 data protection expert, and the medical directors of the three hospitals. The IEC membership did not change during the aforementioned period.

The present study was conducted on the protocols evaluated by the IEC from January 2020 to December 2022, differentiating COVID-19 protocols. The documentation of the projects was submitted to IEC via a single online platform where the researchers uploaded the main required files including synopsis, summary sheet, data collection sheet, protocol, informed consent form, consent form for the processing of personal data (according to the General Data Protection Regulation GDPR UE 679/2016), data processing risk assessment form, declaration on the observational nature of the study, declaration of the non-profit nature of the study, declaration of absence of conflict of interest, researchers’ curricula.

The applications were divided into two group: COVID-19 studies (Group A, GA) and non-COVID-19 studies (Group B, GB).

The following data were evaluated for GA: study type, if observational or not; enrolled participants’ age, minors or not; financial support or not; categories of revised protocols: (a) impact on the population; (b) SARS-CoV2 characteristics; (c) therapeutics (no vaccines); (d) long-term effects, (e) vaccines, (f) diagnostic tests and epidemiology; the distribution of revised protocols by applicants’ medical specialties.

The following data were evaluated for GB: study type, if observational or not; enrolled participants’ age, minors or not; financial support or not; objective of revised protocols: (a) medicinal products, (b) medical devices, (c) diagnostics and therapeutics, (d) other than medicinal products (e.g., supplements, laboratory testing, etc.), (e) biological samples; categories of protocols by setting: clinical, surgical, imaging, efficacy and safety of care, other (e.g., epidemiology); the distribution of revised protocols by applicants’ medical specialties.

Variables’ distributions of the two groups were compared through the Chi-square test (when the expected value for each cell was five or higher) and Fisher’s exact test (when the expected value for each cell was under five) (22). For all tests, significance was set at *α* = 0.001. IBM SPSS Statistic software (version 25) was used. The analysis was carried out for the following variables: study type, if observational or not; enrolled participants’ age, minors or not; financial support or not; distribution of revised protocols by applicants’ medical specialties.

The data are described in aggregate form. The authors did not collect personal identifying information from participants to help protect confidentiality.

In total 1,667 protocols were evaluated by the IEC during the study period: 584 (35.0%: 99 GA and 485 GB) in 2020, 538 (32.2%; 46 GA and 492 GB) in 2021 and 545 (32.6%; 25 GA and 520 GB) in 2021. The number of sessions were increased from 2 to 3 per month, with suspension in August. Three peaks were observed in March 2020 (71 protocols), May 2022 (90 protocols), and September 2022 (67 protocols).

It was observed regarding the study type that most of the studies (1,036 protocols, 62.1%) were observational, while a minority were interventional; more than two-thirds (1,272, 76.3%) had no funding.

**Figure 1:**
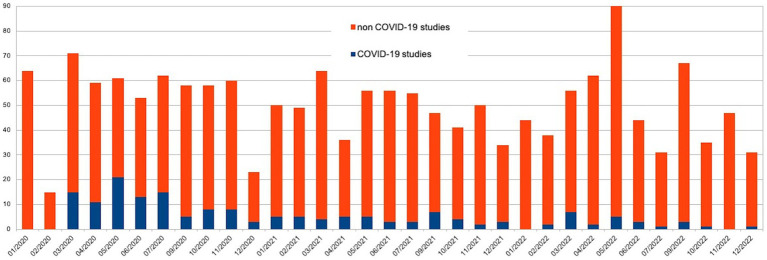
Monthly distribution of the assessed protocols in 2020, 2021 and 2022.

GA and GB included 170 and 1,497 studies, respectively. Figure 1 shows the monthly distribution of the assessed protocols in 2020, 2021 and 2022.

### 3.1 Description of COVID-19 studies evaluated by the IEC

In the context of GA, the peak activity occurred in May 2020, with a total of 21 protocols conducted. The most prolific period was from March 2020 to July 2020. Of the 151 observational studies (88.8%), 164 were not-for-profit (96.4%, see Table 1). Minors were enrolled in only 29 cases (17%).

**Table 1:** Comparative between COVID-19, GA, and non-COVID-19, GB, studies (Jan 2020 to Dec 2022).

N Tot = 1,667	COVID-19 (*N* = 170, 10%)	Non COVID-19 (*N* = 1,497, 90%)
Study design		
Experimental study	19 (11,2%)	612 (40,9%)
Observational study	151 (88,8%)	885 (59.1%)
Funding		
Not for profit	164 (96,4%)	1,108 (74,1%)
For profit	6 (3,6%)	389 (25,9%)
Enrollment of minors		
Yes	29 (17%)	203 (13,5%)
No	141 (83%)	1,294 (86,5%)

**Figure 2:**
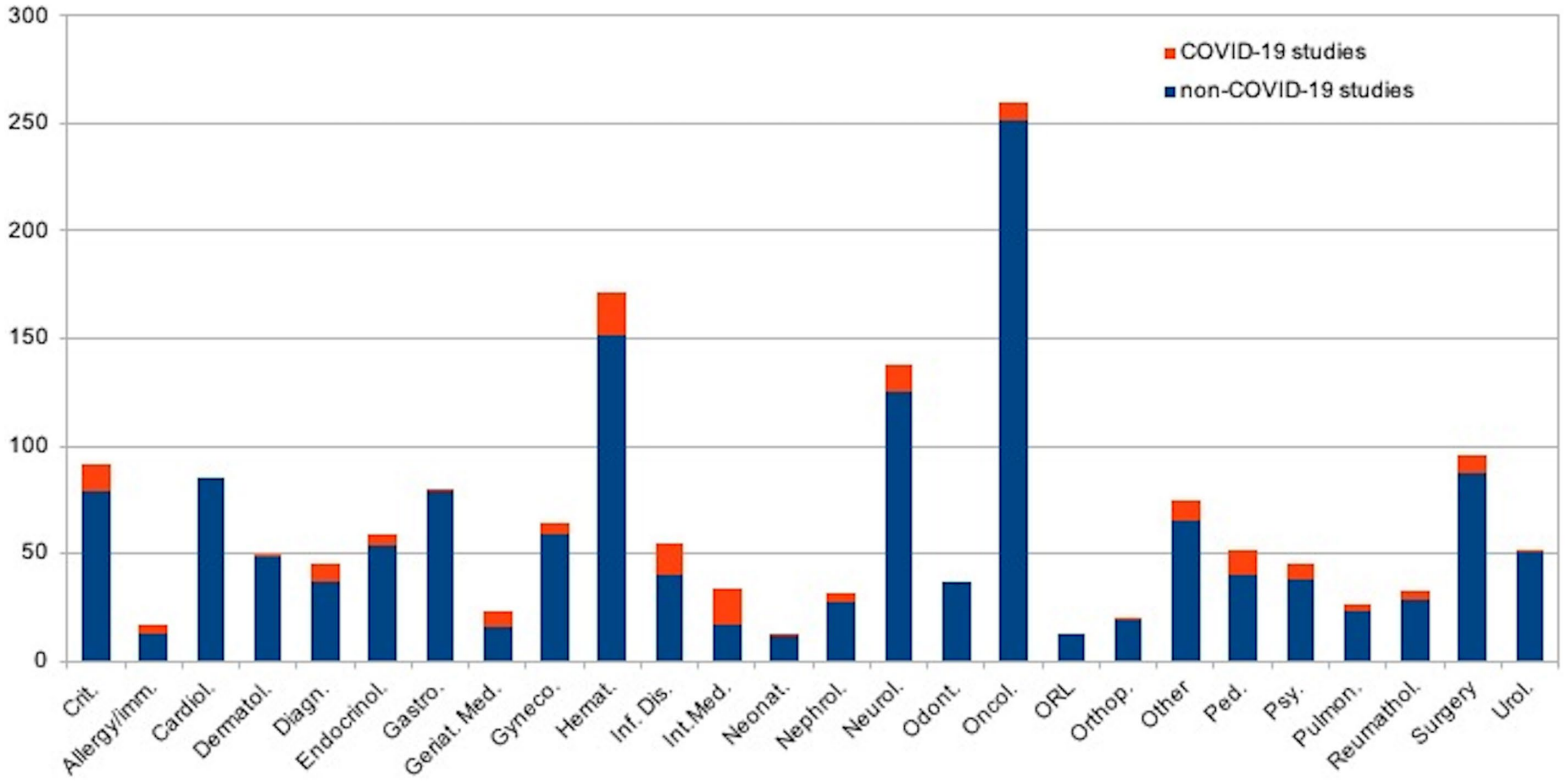
Distribution of revised protocols by applicant’s medical specialities.

Figure 2 shows the distribution of revised protocols by applicants’ medical specialties (embedded with GB studies): 20 Hematology, 17 Internal medicine, 15 Infectious diseases, 13 Critical care medicine; 13 Pediatrics (1 Neonatal-perinatal medicine), 13 Neurology, 9 Surgery, 8 Diagnostic radiology, 8 Oncology, 7 Geriatric medicine, 7 Psychiatry/psychology, 5 Endocrinology, 5 Obstetrics and gynecology, 5 Nephrology, 4 Rheumatology, 4 Allergy and Immunology, 3 Pulmonary diseases, 1 Urology, 1 Dermatology, 1 Gastroenterology, 1 Orthopedics, 10 other specialties (e.g., health management, Ophthalmology, Physical Medicine and Rehabilitation). No studies were promoted by odontologists, cardiologists, or otolaryngologists.

Most of the protocols were related to COVID-19 impact on the population (e.g., on other pathologies and/or therapy) (54, 31.7%), followed by those protocols focused on SARS-CoV2 characteristics (27, 15.8%), therapy (vaccines excluded) (24, 14.1%), long-term effects (24, 14.1%), vaccines (16, 9.4%), diagnosis (16, 9.4%), epidemiology (10, 5.8%). The studies focused on vaccines showed the highest increase from 0 in 2020 to 9 in 2021 and 7 in 2022.

### 3.2 Description of non-COVID-19 studies evaluated by the IEC

For GB, 885 studies were observational (59,1%) and 1,108 were not-for-profit studies (389 were for-profit, 25,9%). Minors were enrolled in 203 of 1,497 cases (13,5%, see Table 1).

Among the objectives of experimental protocols, 362 were on medicinal products, 74 were on medical devices, 64 were therapeutics, 51 were not on drugs (e.g., supplements, laboratory tests), and 60 were on biospecimens.

With regard to the distribution of revised protocols by setting (some studies had double topics), 1785 were clinical, 178 were surgical, 62 were on imaging, 53 were on efficacy and safety of care, and 122 had another topic (e.g., epidemiology, pathology, etc.).

Figure 2 shows the distribution of revised protocols by applicants’ medical specialization (embedded with GA studies): 250 Oncology, 152 Hematology, 125 neurology, 87 surgery, 85 Cardiology, 79 Critical care medicine; 79 Gastroenterology, 59 Obstetrics and gynecology, 54 Endocrinology, 51 Urology, 48 Dermatology, 40 Infectious diseases, 40 Pediatrics, 38 Psychiatry/psychology, 37 Diagnostic radiology, 37 Odontology, 29 Rheumatology, 27 Nephrology, 23 Pulmonary diseases, 19 Orthopedics, 17 Internal medicine, 16 Geriatric medicine, 13 Allergy and Immunology, 13 Otolaryngology, 12 Neonatal-perinatal medicine, 65 other specialties (e.g., health management, Ophthalmology, Physical Medicine and Rehabilitation).

Statistical comparisons (Chi-square test and Fisher test) of frequencies of the GA and GB yielded statistical differences (*p*-value ≥ 0.001) for the variables: observational study (Yes or NOT); for-profit study (YES or NOT); distribution of revised protocols by applicants’ medical specialties. On the contrary, the difference was not statistically significant for the following variable: minors enrolled (YES or NOT).

## 4 Discussion

Healthcare professionals contribute to scientific progress by taking into account three main aims: (i) the protection of the research’s value in encouraging heath progress (rational); (ii) the respect of the subject’s autonomy and integrity (informed consent, risk/benefit balance); (iii) fairness of resource allocation (priorities, social justice) (23).

These targets are closely linked to the classic principles of bioethics outlined in the 1974 Belmont Report by the National Commission for the Protection of Human Subjects of Biomedical and Behavioral Research. These principles were further developed by Beauchamp and Childress in 1979: Autonomy, Beneficence, Non-maleficence, and Justice (6). These fundamental ethical principles address the ethical issues that arise from research involving human subjects.

Ensuring ethical considerations is of primary importance and must underpin any proposal for experimentation on human subjects. Although approval from RECs is essential to conduct research, these committees should be viewed as supporters of the research process rather than obstacles (24).

There is a risk that RECs may be perceived as bureaucratic bodies that could delay the approval process. Instead, there should be a collaborative approach among all professionals involved in the research, as outlined in the Declaration of Helsinki. RECs should actively interact with researchers, offering guidance on ethical issues beyond just informed consent.

It is also essential to recognize the social value of research. The principles of non-maleficence and beneficence must be carefully balanced by RECs when evaluating potential benefits to the community, although achieving this balance is not always straightforward (20).

The COVID-19 pandemic has significantly affected the role of RECs in addressing ethical issues in research. As noted by the World Health Organization, “*In time of a new epidemic outbreak there is a moral obligation to acquire new knowledge as soon as possible, in order to meet public health needs. However, despite the state of emergency, studies should not be conducted without a careful analysis of the risks and quality of the studies*” (25).

As one of the most urgent responses to the COVID-19 pandemic, many European countries established a fast track for the development of research related to the treatment, prevention, and diagnosis of SARS-CoV-2 infections. This included accelerated procedures for authorizing relevant clinical studies (7, 26, 27). These measures facilitated faster patient enrollment in studies and established a single national structure for the approval of clinical studies. However, they also exposed several challenges, including a lack of available staff and infrastructure, difficulties in ensuring adequate quantity and quality of data, issues in coordinating projects among various centers, ineffective organization of regional and national ethical committees, and limited resources (25, 28).

As a result, during the SARS-CoV-2 pandemic, many countries reported a decline in patient enrollment in clinical studies, particularly in cancer control and prevention. This decline was attributed to patients’ fears of infection and the difficulties hospitals faced in managing clinical and therapeutic activities (29, 30).

The COVID-19 pandemic necessitated the adaptation of new enrollment strategies for research institutions (31). Participants in studies were offered alternative methods for involvement, including remote consent and data collection, virtual medical evaluations, and flexible administration of protocol therapy when feasible (32–34). Above all, the privacy and safety of participants remained a top priority.

This study examines the impact of the COVID-19 pandemic on the activities of an Italian REC, considering the characteristics of various research protocols and the distribution of pandemic waves in Italy. The analysis of the three-year period 2020–2022 showed a slight increase in the total number of protocols in 2020 if compared to 2021 and 2022, when the numbers remained relatively steady. During this period, the number of COVID-related studies decreased, while there was a proportional increase in non-COVID-related studies. This trend suggests that the organizational structure of the Independent EC successfully adapted its recruitment strategies in response to the challenges presented by the pandemic, despite encountering obstacles in the approval process (35–37).

The dramatic increase in medical research during the pandemic raised concerns about the potential lowering of research standards, as several authors have pointed out (38). De Man et al. noted the rise of online research due to challenges in accessing participants in person and time constraints, which led to risks of selection bias and the use of non-validated scales (39).

In this context, the role of EC was crucial in maintaining scientific validity and ensuring the safety of research. This safeguarding should never be compromised due to time pressure or, even worse, economic considerations. Other authors, including Sisk et al., confirmed this view, highlighting that professionals and researchers within the United States Institutional Review Boards faced numerous challenges when conducting COVID-19 research in the early stages of the pandemic. These challenges included issues related to policy, biases and misperceptions, conflicts both within and between institutions, risks of harm, and the overall pressure of the pandemic (32). These issues were perceived as “barriers,” particularly in the early months, which could be addressed through a comprehensive reorganization of research personnel and structures (28).

In our study, most of the COVID-19-related protocols focused on the pandemic’s impact on the population, particularly regarding other diseases and therapies. This aligns with existing literature that describes a decline in enrollment for clinical studies, especially those related to cancer control and prevention, during the pandemic. This decline was significant, as limited access to healthcare facilities, resource constraints, and refusals or losses of follow-up were common due to pandemic restrictions (29, 30).

We identified three key variables that differed between the COVID-19-related and non-COVID-19-related groups: the observational nature of the study, funding from for-profit sponsors, and the distribution of protocols based on applicants’ medical specialties.

Firstly, the observational type of study showed a significant increase in the COVID-19-related group. It was noted that a higher proportion of COVID-related studies were observational compared to non-COVID-related studies, which were predominantly experimental. The present study could not determine a definitive reason for this difference due to its retrospective and observational nature. However, it can be suggested that the initial lack of information regarding SARS-CoV-2 led researchers to rely on the limited data available at the time, which often came from retrospective analyses of observational and non-experimental case studies. Indeed, it took a few months for research groups to start implementing studies on the topic during the pandemic (37).

With regard to the second variable, which is funding from for-profit sponsors, there were fewer for-profit studies in both groups. In COVID-19-related studies, the prevalence of unfunded research can be attributed to the pressing need for rapid scientific advancements focused on public health rather than commercial interests. Academic institutions, public hospitals, and research centres spearheaded numerous such studies with the primary objective of comprehending the virus, enhancing patient care, and devising effective prevention and treatment strategies, rather than pursuing profit maximization. This aligns with the broader global trend during the emergency, where collaboration and knowledge sharing were prioritized over market-driven research.

When examining the distribution of revised protocols by applicants’ medical specialties, it was noted that critical care medicine, infectious diseases, diagnostic radiology, hematology, and geriatric medicine were proportionally more represented in the GA than in the GB. This observation is not unexpected, as these disciplines were at the forefront of diagnosing and treating COVID-19. Notably, the findings related to infectious diseases and diagnostic radiology are particularly significant (37). The high number of COVID-related studies promoted within critical care highlights the increasing focus of researchers on providing healthcare support for critically ill patients from the very beginning of the pandemic. Similarly, the data surrounding hematology’s contribution is noteworthy due to the significant impact of SARS-CoV-2 infection on the hematological system over time (40).

The difference in the enrollment of minors between groups GA and GB was not statistically significant. This may be related to the ethical issues surrounding the enrollment of minors in research protocols, which require careful planning, coordination, and efforts to minimize the risk of exposing them to excessive research dangers. It is important to note that minors cannot provide informed consent unless they are deemed capable of making such decisions themselves (for example, emancipated or mature minors). Additionally, parental consent is required for children’s participation in research. This consideration is especially critical in COVID-19-related studies, particularly those involving vaccines, as underage subjects began to be infected in increasing numbers as the pandemic progressed, unlike in the early stages when few minors were affected (41).

The main limitations of this manuscript stem from the lack of available data concerning the activity of the IEC in 2019, the year it was established, during which it also had a different membership. As a result, it was not possible to compare the IEC’s activities between the pre-pandemic and pandemic eras. This study can only be used to describe and evaluate the impact the pandemic had on the IEC’s activities and the studies assessed over the months, specifically comparing COVID-19 protocols with non-COVID-19 protocols.

During the COVID-19 pandemic, although it was a global event, not all countries implemented the same restrictions, whether in daily life or in the research field. This highlights the World Health Organization’s role in coordinating pandemic responses on a global scale.

The analysis of numerous projects conducted by the Institutional ECs underscored the importance of having a national committee to uphold ethical principles, which are essential in research involving human participants. The EC overview system should be seen as a beneficial process rather than a barrier to scientific progress, enhancing safety and protecting participants’ rights.

There is a strong need for the standardization of review procedures, particularly during global emergencies, to address the variations in restrictions across different nations. This standard approach to research should be adopted for future emergencies.

## Data Availability

The raw data supporting the conclusions of this article will be made available by the authors, without undue reservation.
